# Draft Genome Sequence of *Bacillus amyloliquefaciens* B-1895

**DOI:** 10.1128/genomeA.00633-14

**Published:** 2014-06-19

**Authors:** Andrey V. Karlyshev, Vyacheslav G. Melnikov, Vladimir A. Chistyakov

**Affiliations:** a School of Life Sciences, Faculty of Science, Engineering and Computing, Kingston University, Kingston upon Thames, United Kingdom; b International Science and Technology Center, Moscow, Russia; c Genome Variability Department, Research Institute of Biology, Southern Federal University, Rostov on Don, Russia

## Abstract

In this report, we present a draft genome sequence of *Bacillus amyloliquefaciens* strain B-1895. Comparison with the genome of a reference strain demonstrated similar overall organization, as well as differences involving large gene clusters.

## GENOME ANNOUNCEMENT

*Bacillus* sp. strain B-1895, with enhanced proteolytic activity, was isolated and characterized at the Research Institute for Genetics and Selection of Industrial Microorganisms, Moscow, Russia, in 1979. Although it was originally identified as *B. subtilis*, our current study demonstrated that this strain is in fact *B. amyloliquefaciens*. Strain B-1895 is commercially used as a probiotic in the fish industry (1), particularly for rearing Azov Sea basin “shemaya” (royal fish) variety *Alburnus leobergi*, replacing the use of antibiotics.

Genome sequencing reads were produced by an IonTorrent PGM and 314v2 chip. The original assembly using the IonTorrent assembler plugin resulted in a large number (407) of relatively small (up to 92 kb) contigs with the total size of 4,250,427 bp. In order to improve the assembly, the reads were mapped onto the *B. amyloliquefaciens* LFB112 genome sequence using CLC Genomics Workbench software (version 7.0), which allowed extraction of 76 consensus sequences of up to 430 kb in size. The unmapped reads were assembled *de novo* and added to consensus sequences to produce a total of 186 contigs.

The genome size of strain *B. amyloliquefaciens* B-1895 (4,107,280 bp, 25.59× genome coverage) and G+C content (46.2%) are in a full agreement with the respective figures for the complete genome sequences of other strains of this species (3.89 to 4.24 Mb and 46.1 to 46.7%, respectively).

Genome annotation using the RAST server (2) revealed the presence of 4,118 protein-encoding genes, including those responsible for the biosynthesis of bacitracin-like antibiotics, capsule/exopolysaccaride/teichoic acid, and N-linked glycoproteins (PglC, PglE, and PglF homologues). A beta-lactamase gene, the genes responsible for the biosynthesis of streptothricin acetyltransferase (3), and genes for resistance to fluoroquinolone and tetracycline antibiotics were found. A toxin-antitoxin (MazEF-like) system involved in programmed cell death is present in *B. amyloliquefaciens* B-1895. Analysis of the derived amino acid sequences using a BLASTp search and the non-redundant amino acid sequence database revealed the highest level of similarity with the proteins of *B. amyloliquefaciens* LFB112. The two strains have very similar genome organization, despite the presence of a large number of single base pair polymorphisms in most genes. The strains share a large gene cluster (over 12 kb) involved in the biosynthesis of teichuronic acid. A large (ca. 35 kb) gene cluster containing genes encoding gramicidin/bacitracin synthetases and a set of genes involved in modification of/resistance to lantibiotics are present in the reference but not in the test strain. However, the test strain does have the potential to produce various types of cyclic peptide antibiotics due to the presence of a number of genes encoding other peptide synthetases. The reference strain has three major facilitator transporter genes missing in the test strain.

The discovered differences between the genomes of the test and reference strains and the presence of transposase-encoding genes and prophages suggest the mechanisms involved in genome rearrangements in these bacteria. The availability of the *B. amyloliquefaciens* B-1895 genome sequence will assist in understanding the specific properties of this strain.

### Nucleotide sequence accession numbers.

This whole-genome shotgun project has been deposited at DDBJ/EMBL/GenBank under the accession no. JMEG00000000. The version described in this paper is version JMEG01000000.
